# Explainable CT-based deep learning model for predicting hematoma expansion including intraventricular hemorrhage growth

**DOI:** 10.1016/j.isci.2025.112888

**Published:** 2025-06-13

**Authors:** Xianjing Zhao, Zhengxiang Zhang, Juntao Shui, Hui Xu, Yulong Yang, Lequn Zhu, Lei Chen, Shixin Chang, Chunzhong Du, Zhenwei Yao, Xiangming Fang, Lei Shi

**Affiliations:** 1 Department of Radiology, Zhejiang Cancer Hospital, Hangzhou, Zhejiang, China; 2 Hangzhou Institute of Medicine (HIM), Chinese Academy of Sciences, Hangzhou, Zhejiang, China; 3 Department of Neurology, The First Affiliated Hospital of Zhejiang Chinese Medical University (Zhejiang Provincial Hospital of Chinese Medicine), Hangzhou, Zhejiang, China; 4 Department of Radiology, The First People’s Hospital of Hangzhou Lin’an District, Hangzhou, Zhejiang, China; 5 Department of Radiology, Yueyang Hospital of Integrated Traditional Chinese and Western Medicine Shanghai University of Traditional Chinese Medicine, Shanghai, China; 6 Department of Rehabilitation Medicine, Yueyang Hospital of Integrated Traditional Chinese and Western Medicine, Shanghai University of Traditional Chinese Medicine, Shanghai, China; 7 Department of Radiology, Huashan Hospital, Fudan University, Shanghai, China; 8 Department of Medical Imaging, The Affiliated Wuxi People’s Hospital of Nanjing Medical University, Wuxi, Jiangsu Province, China

**Keywords:** Medical imaging, Artificial intelligence

## Abstract

Hematoma expansion (HE), including intraventricular hemorrhage (IVH) growth, significantly affects outcomes in patients with intracerebral hemorrhage (ICH). This study aimed to develop, validate, and interpret a deep learning model, HENet, for predicting three definitions of HE. Using CT scans and clinical data from 718 ICH patients across three hospitals, the multicenter retrospective study focused on revised hematoma expansion (RHE) definitions 1 and 2, and conventional HE (CHE). HENet’s performance was compared with 2D models and physician predictions using two external validation sets. Results showed that HENet achieved high AUC values for RHE1, RHE2, and CHE predictions, surpassing physicians’ predictions and 2D models in net reclassification index and integrated discrimination index for RHE1 and RHE2 outcomes. The Grad-CAM technique provided visual insights into the model’s decision-making process. These findings suggest that integrating HENet into clinical practice could improve prediction accuracy and patient outcomes in ICH cases.

## Highlights

• HENet predicts hematoma expansion with superior performance in ICH patients
• HENet outperforms physicians in predicting revised hematoma expansion definitions
• Grad-CAM highlights regions that influence HENet’s hematoma expansion predictions

## Graphical abstract

**Figure:**
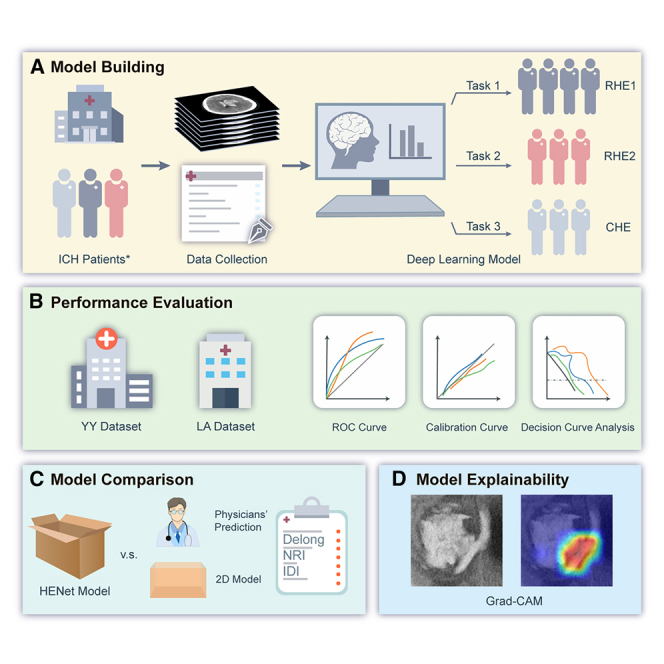

## Introduction

Intracerebral hemorrhage (ICH) is a severe type of stroke characterized by bleeding within the cerebral tissue.^1^ Hematoma expansion (HE) serves as a promising therapeutic target and surrogate endpoint focusing on acute ICH cases.^2^^,^^3^ Intraventricular hemorrhage (IVH) growth is a consequential complication of ICH that exerts a substantial influence on patient outcomes.^4^ However, conventional HE (CHE) definitions do not account for this aspect. Recently, revised HE (RHE) definitions have been proposed to include IVH growth.^5^ These revised definitions demonstrate superior predictive capabilities for 90-day functional outcomes compared to the CHE definitions.^6^

The identification of patients at the highest risk of HE is crucial for improving the evaluation of treatment effectiveness in future trials.^7^ Consequently, extensive research efforts by researchers globally have been dedicated to investigating the predictors of HE. In addition to age, Glasgow Coma Scale (GCS), onset to CT time, subarachnoid hemorrhage (SAH), hematoma volume, and location, non-contrast computed tomography (NCCT) markers have also been identified as predictive factors for HE.^8^^,^^9^ Researchers have developed multiple predictive scores based on these factors, but there is no clear consensus on which tool has the best predictive ability.^7^ Furthermore, it is still unknown whether these scores can accurately predict RHE. The lack of reliable predictors for HE hinders early detection by physicians and subsequent proactive intervention. Therefore, prospectively identifying high-risk patients with HE remains a priority.^10^

Deep learning, as a field of technological advancement, has the potential to revolutionize current diagnostic and management strategies by utilizing its capabilities in HE predictions.^8^ Some studies have attempted to predict the risk of HE using 2D deep learning techniques.^11^^,^^12^^,^^13^^,^^14^ However, these models suffer from several limitations. First, these studies have not considered RHE definition. Second, several studies have been conducted in single-center settings and lack validation in multiple centers.^12^^,^^14^ Additionally, the lack of explainability is a drawback of deep-learning-based methods.

**Figure 1:**
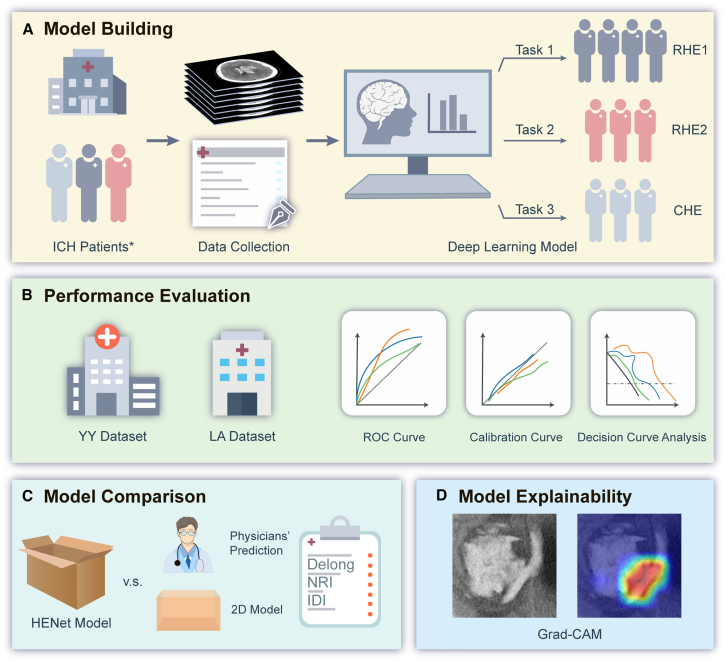
Experimental process diagram (A) Model building. (B) Performance evaluation. (C) Model comparison. (D) Model explainability. CHE, conventional hematoma expansion; Grad-CAM, gradient-weighted Class Activation Mapping; IDI, integrated discrimination improvement; NRI, net reclassification improvement; RHE1, revised hematoma expansion definition one; RHE2, revised hematoma expansion definition two; ROC, receiver operating characteristic. ∗The clinical and imaging information included in the model building came from ICH patients in the HS dataset.

**Figure 2:**
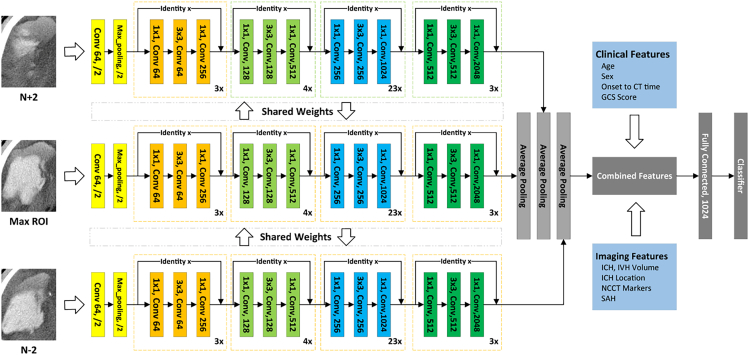
Architecture of HENet model Conv, convolution; GCS, Glasgow Coma Scale; ICH, intracerebral hemorrhage; IVH, intraventricular extension of intracerebral hemorrhage; SAH, subarachnoid hemorrhage.

Therefore, the aim of this research was to develop and externally validate a novel 2.5D deep convolutional neural network (CNN) model, referred to as HENet model, for predicting three distinct definitions of HE, namely: RHE definition 1 (ICH >6 mL or 33% or any IVH expansion), RHE definition 2 (ICH >6 mL or 33% or IVH >1 mL), and CHE (ICH >6 mL or 33%). Subsequently, HENet model was compared with 2D models and physicians’ predictive results in two external validation sets. Finally, the study also employs Gradient-weighted Class Activation Mapping (Grad-CAM) and utilizes gradients and feedforward responses to generate attention maps for visual explanation. A detailed experimental process diagram is illustrated in Figure 1. The overall architecture is illustrated in Figure 2.

## Results

### Characteristics of study datasets

The patient and hematoma characteristics of patients in the HS, YY, and LA datasets are summarized in Table 1. The HS dataset included 522 subjects, with a median age of 62 years, while the YY and LA datasets included 100 and 96 subjects, with median ages of 57 and 68 years, respectively. Majority of subjects across all datasets are males. Age, time from onset to CT scan, and GCS scores show significant differences among the datasets (*p* < 0.001). In terms of ICH location, deep and lobar locations are most prevalent. Significant variations were observed in ICH location (*p* = 0.002), ICH volume (*p* = 0.038), IVH volume (*p* = 0.033), and the presence of the RHE1 (*p* = 0.006). No significant differences exist regarding NCCT markers and the presence of SAH, RHE2, and CHE.

**Table 1:** Baseline characteristics of the HS, YY, and LA datasets

Characteristics	HS dataset (*n* = 522)	YY dataset (*n* = 100)	LA dataset (*n* = 96)	*p* Value
Age^a^	62 (49–71)	57 (47–66)	68 (60–76)	<0.001^b^
Sex				0.079
Male	395 (76)	67 (67)	65 (68)	
Female	127 (24)	33 (33)	31 (32)	
Onset to CT time^a^	4 (3–5)	2 (1–4)	3 (2–5)	<0.001^b^
GCS score^a^	15 (11–15)	15 (15–15)	14 (12–15)	<0.001^b^
ICH location				0.002^b^
Deep	317 (61)	74 (74)	56 (58)	
Lobar	141 (27)	20 (20)	27 (28)	
Brain stem	37 (7)	1 (1)	1 (1)	
Cerebellum	27 (5)	5 (5)	12 (12)	
ICH volume^a^	20 (9–45)	22 (11–43)	14 (7–34)	0.038^b^
IVH volume^a^^,^^c^	8 (3–17)	8 (2–22)	3 (2–6)	0.033^b^
Heterogeneous density	145 (28)	28 (28)	28 (29)	0.962
Swirl sign	334 (64)	64 (64)	71 (74)	0.161
Hypodensity	298 (57)	62 (62)	63 (66)	0.235
Black hole sign	211 (40)	42 (42)	46 (48)	0.391
Blend sign	36 (7)	9 (9)	6 (6)	0.71
Fluid level	43 (8)	2 (2)	5 (5)	0.062
Irregular shape	242 (46)	42 (42)	34 (35)	0.124
Island sign	125 (24)	22 (22)	17 (18)	0.399
Satellite sign	248 (48)	43 (43)	41 (43)	0.537
SAH	56 (11)	4 (4)	12 (12)	0.084
CHE	125 (24)	31 (31)	27 (28)	0.272
RHE1	414 (79)	68 (68)	83 (86)	0.006^b^
RHE2	177 (34)	42 (42)	31 (32)	0.255

Except where indicated, data are numbers of patients, with percentages in parentheses.

CHE, conventional hematoma expansion; GCS, Glasgow Coma Scale; ICH, intracerebral hemorrhage; IVH, intraventricular extension of intracerebral hemorrhage; RHE1, revised hematoma expansion definition one; RHE2, revised hematoma expansion definition two; SAH, subarachnoid hemorrhage.

a Data are medians, with interquartile ranges in parentheses.

b Statistical significance; *p* < 0.05.

c IVH was presented in 241 patients in the HS dataset, 43 patients in the YY dataset, and 28 patients in the LA dataset.

### Model performance

The HENet model exhibits significant predictive power in the context of HE, as demonstrated by its performance across various metrics. For RHE1 prediction, HENet model achieved an AUC of 0.882 and 0.854 for the YY and LA datasets respectively, with high accuracy scores of 0.870 and 0.844. The model’s sensitivity was particularly notable, peaking at 0.956 for the YY dataset. In predicting RHE2, HENet model maintained consistent positive predictive value (PPV) despite variations in sensitivity and specificity between datasets, indicating a reliable prediction of true positives. For the CHE, while values varied across the board, the model’s negative predictive value (NPV) remained high, underscoring its robust ability to correctly identify non-expanding hematomas. These findings, which are displayed in both Table 2, Table S7 and Figure S2, reinforce the efficacy and reliability of the HENet model in predicting HE.

**Table 2:** Performance of HENet model in predicting hematoma expansion

Outcome	Dataset	AUC	95% CI	Accuracy	Sensitivity	Specificity	PPV	NPV
RHE1	YY	0.882	0.808–0.956	0.870	0.956	0.688	0.867	0.880
	LA	0.854	0.739–0.970	0.844	0.843	0.846	0.972	0.458
RHE2	YY	0.728	0.630–0.826	0.680	0.857	0.552	0.581	0.842
	LA	0.811	0.724–0.900	0.750	0.677	0.785	0.600	0.836
CHE	YY	0.712	0.599–0.825	0.620	0.806	0.536	0.439	0.860
	LA	0.716	0.591–0.842	0.750	0.556	0.826	0.556	0.826

AUC, area under the curve; CHE, conventional hematoma expansion; NPV, negative predictive value; PPV, positive predictive value; RHE1, revised hematoma expansion definition one; RHE2, revised hematoma expansion definition two.

The calibration curve analysis for the HENet model demonstrated reasonable agreement between predicted and actual probabilities across both datasets for all definitions of HE (Figure S3). Within the YY dataset, the decision curves revealed that the HENet Model outperformed both the treat-all-patients and treat-none schemes in terms of net benefits when the threshold probability ranged from 30% to 95% for RHE1, 30% to 70% for RHE2, and 20% to 65% for CHE. Similarly, the LA dataset showed that the HENet Model could exceed the net benefits of both schemes when the threshold probability was within 65%–95% for RHE1, 0%–100% for RHE2, and 18%–65% for CHE (Figure S4).

### Model comparison

Table S2 illustrates the intraclass correlation coefficient of HE predicted by two physicians. The performance of physicians’ predictions and the 2D model are individually delineated in Tables S3 and S4, respectively. The ROC curves along with calibration and decision curves for physicians’ predictions and the 2D model can be found in Figures S2, S3, and S4, respectively.

From the comparative analysis in Table S5, the HENet model significantly outperformed physicians’ predictions in NRI and IDI for RHE1 and RHE2 outcomes across both datasets. Although HENet showed higher values for CHE outcome, these differences were not statistically significant, indicating comparable performance with physician’s predictions for this specific outcome.

Table S6 displayed a comparative analysis of the HENet and 2D models regarding HE. For both RHE1 and RHE2 outcomes, significant rises were detected in NRI and IDI values across datasets, validated by *p*-values less than 0.001. Although increases in AUC values were recorded, these did not meet statistical significance with *p*-values over 0.05. For the CHE outcome in the YY dataset, all measures displayed significant increases (*p* < 0.001 and *p* = 0.006). Third, pertaining to the LA dataset, although there was a decrease in AUC values exhibited by the HENet model compared to the 2D model in predicting CHE, this reduction was not statistically significant.

Table S8 displays the performance of the 3D model in predicting HE. Table S9 presented the comparative analysis between the HENet and the fully 3D model. Across multiple outcomes (RHE1, RHE2, and CHE) and datasets (YY and LA), the HENet model consistently demonstrated favorable net reclassification improvement (NRI) and integrated discrimination improvement (IDI) metrics. For the RHE1 outcome, the HENet model showed non-significant AUC increases in both the YY dataset (*p* = 0.245) and the LA dataset (*p* = 0.18). For RHE2, significant AUC improvements were observed in both the YY dataset (*p* < 0.001) and the LA dataset (*p* < 0.001). For the CHE outcome, a significant AUC improvement was seen in the YY dataset (*p* = 0.023), while the increase in the LA dataset was not significant (*p* = 0.222).

Table S10 illustrates the predictive performance of the clinical-only ExtraTrees model for HE. Table S11 presents a comparative analysis of the HENet model versus the ExtraTrees model in predicting HE. For the RHE1 outcome, the HENet model demonstrated significant AUC improvements in both the YY dataset (*p* < 0.001) and the LA dataset (*p* = 0.001). For RHE2, significant AUC increases were observed in the YY dataset (*p* = 0.001), while the increase in the LA dataset was not statistically significant (*p* = 0.127). For the CHE outcome, significant AUC improvement was seen in the YY dataset (*p* = 0.001), whereas the increase in the LA dataset was not significant (*p* = 0.421).

### Model interpretation

**Figure 3:**
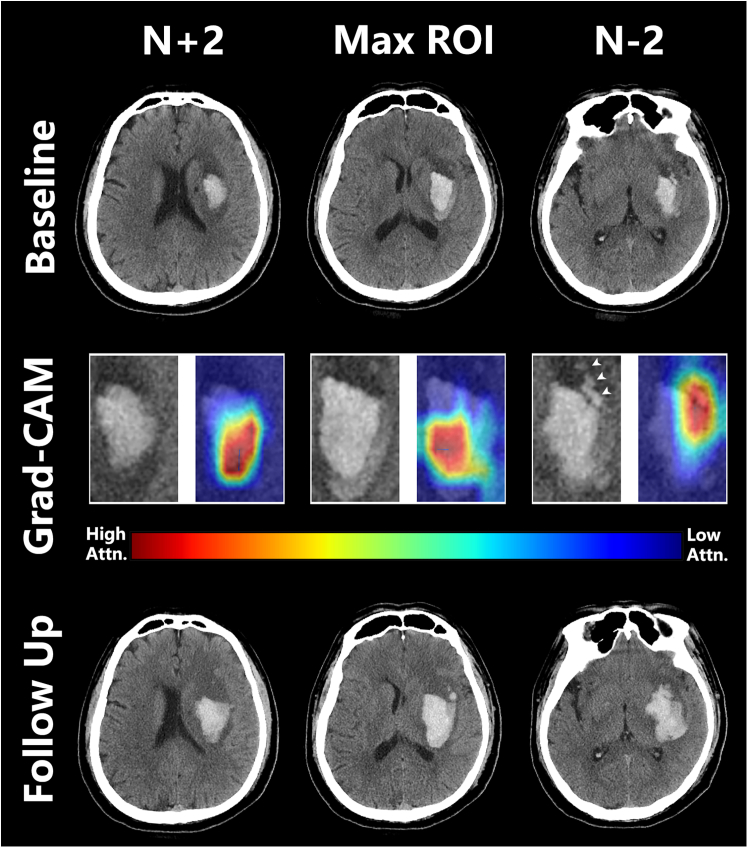
Model explainability with Grad-CAM in a patient with intracerebral hemorrhage at the left basal ganglia The first row depicts the baseline CT images of the patient. In the second panel, individual-specific explanations are displayed as high-resolution attention heatmaps using attention-based interpretability. Regions of heightened attention (highlighted in red) within the heatmap correspond to morphological characteristics that contribute to the model’s predicted hematoma expansion. White arrows represent the island sign. The bottom row presents the follow-up CT images, revealing an enlargement of the patient’s intracerebral hemorrhage volume.

**Figure 4:**
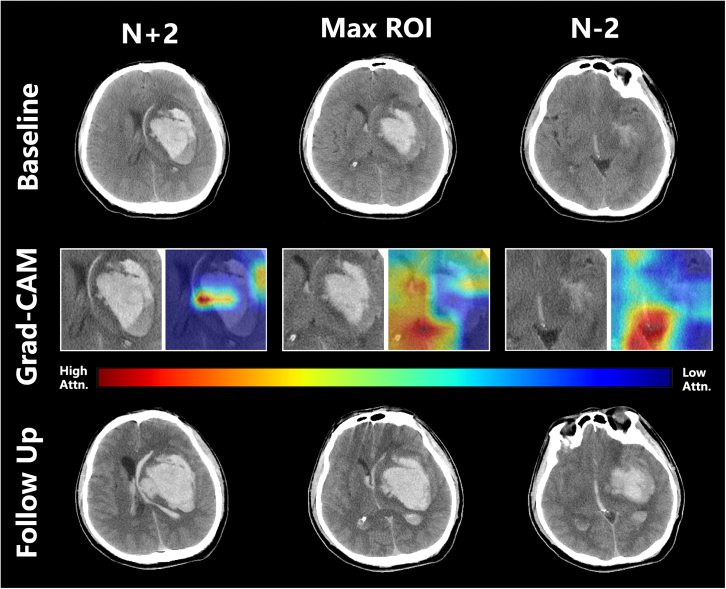
Model explainability with Grad-CAM in a patient with intracerebral hemorrhage at the left basal ganglia and intraventricular hemorrhage extension The first row displays the baseline CT images of the patient. In the second panel, localized explanations for each patient are presented as high-resolution attention heatmaps utilizing interpretability based on attention. Regions exhibiting elevated attention (indicated in red) within the heatmap align with morphological characteristics that contribute to the model’s predicted expansion of the hematoma. The bottom row presents the follow-up CT images, indicating the growth of both the patient’s intracerebral hemorrhage and intraventricular hemorrhage extension.

The Grad-CAM technique was applied to visualize the activation of the last convolutional layer for HE prediction. By overlaying the last convolutional layer transparent, the regions within the input image that contribute the most to the HE prediction were demonstrated. The colors on the heatmap represent the degree of contribution of each region to the prediction result. Red regions indicate positive influence (evidence), meaning that features in these areas are more likely to lead the model toward predicting a certain category. Blue regions indicate negative influence (counter-evidence), suggesting that features in these areas are more likely to discourage the model from predicting a certain category. This visualization provides valuable insights into the decision-making process of the model, without requiring complex architectural changes or retraining of the model (Figures 3 and 4).

## Discussion

In this study, we introduced a deep learning model for HE prediction, identified as the HENet model. The HENet model amalgamates the capability of 2D transfer learning and maximizes the capture of 3D spatial information by extracting lesion context frames. Moreover, the model integrates clinical features in an end-to-end manner. The performance of this model has been validated in two external validation cohorts in terms of discrimination, calibration curve, and clinical utility, achieving satisfactory results in predicting RHE and CHE. Regarding model interpretability, the HENet model possesses superior interpretability, employing the Grad-CAM technique to visualize the contributions of different regions within input images to the HE prediction. This offers invaluable insights into the decision-making process of the model, augmenting our comprehension of its function without requiring complex modifications or retraining of the model.

While previous studies using deep learning models have predominantly focused on predicting CHE, RHE has received comparatively less attention.^11^^,^^12^^,^^13^^,^^14^^,^^15^ For example, Tran et al. and Guo et al. centered on CHE prediction, whereas our study explicitly targets both RHE and CHE, offering a more comprehensive outcome assessment.^11^^,^^15^ Our research strives to fill this gap by concentrating on RHE prediction. The HENet model demonstrated a robust performance in predicting RHE1, with AUCs of 0.882 and 0.854 for the YY and LA datasets respectively. It also performed reasonably well in predicting RHE2, achieving AUCs of 0.728 and 0.811 for the two datasets respectively. Notably, the HENet model significantly outperformed clinicians’ predictions for RHE outcomes across both cohorts. When juxtaposed with 2D models, HENet registered impressive improvements in NRI and IDI values for RHE outcomes, underscored by significant *p*-values. While some papers did not externally validate their 2D models,^12^^,^^14^ our study conducted external validation on two datasets for these models. As for predicting CHE outcomes, the HENet model displayed reasonable performance. The AUC was 0.712 for the YY dataset, and the AUC was 0.716 for the LA dataset. In contrast to Yu et al., which focused on hematoma segmentation, our work emphasizes predictive modeling, combining imaging with clinical data for outcome forecasting.^16^ These findings further emphasize that while clinical features contribute to overall performance, the imaging component remains a key factor in improving predictive accuracy. Including imaging information is critical not only for immediate predictive gains but also for identifying potential directions to enhance future model development.

Currently, the pathophysiological mechanisms underlying HE remain unclear. Miller Fisher proposed an alternative “avalanche” model, suggesting that the initial hematoma displaces and ruptures surrounding vessels, resulting in additional bleeding.^17^ Our model’s interpretable results utilizing Grad-CAM (Figure 3) indicate that it concentrates on the periphery of the hematoma, lending support to the “avalanche” model viewpoint. This locational emphasis identified by our model mirrors findings from Trans et al., who employed a deep learning model predicting hematoma growth without accounting for the expansion of IVH.^15^ Moreover, at the N+2 layer of Grad-CAM in Figure 3, the model exhibits attention toward multifocal minor hemorrhages surrounding the hematoma (island sign). One possible underlying cause of the island sign is that it represents multifocal active bleeding from several ruptured small arteries, which is also consistent with the “avalanche” model.^18^ Furthermore, for ICH patients with expanding IVH, Grad-CAM analysis (Figure 4) reveals that the HENet model particularly highlights the ventricular region. This observation indirectly reinforces the idea that the HENet model perceives IVH growth as a consequential complication of ICH significantly affecting patient outcomes. The analysis using Grad-CAM unveils significant instability of ICH and IVH regions, suggesting a predisposition toward active bleeding and further expansion in these areas. This insight offers a crucial perspective on our understanding of the mechanisms behind HE.

Our study introduces a novel 2.5D deep learning model that incorporates robust performance, high interpretability, and clinical usability. These advancements play a crucial role in enhancing the prediction of HE risk and assisting in understanding the mechanism of the deep learning model in predicting HE. Given the results of this study, we believe that the HENet model will have a positive impact on clinical practice: Deep learning technology could be used as clinical decision support systems through incorporation into electronic medical records and PACS systems. HENet is capable of accurately identifying patients at risk of HE, enabling timely intervention and transfer to intensive care units for close monitoring and active management. Clinician decisions enhanced by deep learning have the potential to improve outcomes in patients with ICH.

### Limitations of the study

This research exhibits a number of limitations. First, it was conducted as a retrospective study, highlighting the need for future prospective studies with larger patient cohorts. Second, the lack of HE prediction scores prevented a direct performance comparison between the HENet model in this study, such as utilizing metrics like BAT score and BRAIN score.^19^^,^^20^ It is recommended that future prospective studies include the collection of such data to further expand upon and validate our findings. Third, the study opted for a 2.5D model instead of a 3D model. The 2.5D model demonstrates higher computational efficiency, enabling rapid processing of medical image data and offering improved operability and practicality in clinical applications. Additionally, the 2.5D model provides a larger parameter adjustment space, combining the characteristics of both 2D and 3D models, allowing for more parameter adjustments to further enhance the model’s performance and applicability. Fourth, the CT lesions in this study were not automatically segmented, as manual segmentation had been performed on these patients previously. Leveraging the professional knowledge and experience of physicians, manual segmentation yielded the most reliable results.

### Conclusion

The model developed in this study, HENet, provides an innovative and reliable tool that incorporates robust performance, high interpretability, and clinical usability. These advancements play a crucial role in enhancing the prediction of HE risk and assisting in understanding the mechanism of the deep learning model in predicting HE.

## Resource availability

### Lead contact

Further information and requests for resources and reagents should be directed to and will be fulfilled by the lead contact, Lei Shi (shilei@zjcc.org.cn).

### Materials availability

This study did not generate unique reagents.

### Data and code availability

• All data reported in this paper will be shared by the lead contact upon request.
• All original code has been deposited at Github at [https://github.com/xzhao4/HENet] and is publicly available as of the date of publication.
• Any additional information required to reanalyze the data reported in this paper is available from the lead contact upon request.

## Acknowledgments

This work was supported by 10.13039/501100004731Zhejiang Provincial Natural Science Foundation of China (LQ24H220001), Zhejiang Province Medical and Health Science and Technology Program (2024KY786), National Nature Cultivation Fund of Zhejiang Cancer Hospital (PY2022045), Zhejiang Provincial Inheritance Studio of Prominent Chinese Medicine Expert Prof. Qiu Changlin (GZS2021007), and Wuxi Medical Innovation Team Program (CXTD002). The experimental flow chart was drawn by Figdraw (www.figdraw.com). We thank OnekeyAI platform for the help on the code implementation.

## Author contributions

X.Z., Z.Z., J.S., H.X., Y.Y., Z.Y., X.F., and L.S.: Conceptualization; data curation; formal analysis; funding acquisition; investigation; methodology; project administration; resources; software; supervision; writing – original draft. L.Z., L.C., S.C., and C.D.: Data curation; writing – review and editing.

## Declaration of interests

The authors declare no competing interests.

## STAR★Methods

### Key resources table

**Table:** 

REAGENT or RESOURCE	SOURCE	IDENTIFIER
**Software and algorithms**		

ITK-SNAP (version 3.8.0)	ITK-SNAP software	http://www.itksnap.org/
R (version 3.6.1)	R software	https://www.r-project.org
Python (version 3.11.3)	Python Software Foundation	https://www.python.org/
PyTorch (version 1.8.1)	PyTorch software	https://pytorch.org/
Onekey (version 2.5.3)	OnekeyAI-Platform	https://github.com/OnekeyAI-Platform/onekey

### Experimental model and study participant details

The data of patients diagnosed with ICH were retrospectively collected for the construction and testing of the deep learning model. This collection comprised three datasets, each derived from a different hospital: a training set from HS Hospital (between February 2014 and December 2019; HS dataset), and two independent external testing datasets respectively collected from YY Hospital (between June 2017 and June 2021; YY dataset) and LA Hospital (between August 2022 and August 2023; LA dataset), respectively. Ethics approvals were obtained by the respective institutional research ethics review boards. Informed consent was waived because of the retrospective nature of the study. Eligible patients were those who underwent both baseline (<6 h from clinical onset) and 24 h follow-up CT examinations. Patients’ exclusion criteria were as follows: (a) surgery before follow-up CT examinations and (b) incomplete examinations or severe artifacts. The detailed patient recruitment process is shown in Figure S1.

CT scans were acquired with six different CT scanners from four various vendors. Details pertaining to the CT scan acquisition can be found in Table S1. Manual segmentation was conducted to measure the ICH volume and IVH volume. NCCT markers were manually annotated. Clinical data, including age, sex, the time interval from symptom onset to baseline CT, and GCS score upon admission, were collected.

Two physicians independently estimated the patient’s risk of three different definitions of HE (RHE1, RHE2, CHE), based on the baseline clinical and imaging data presented in this study.

### Method details

#### Image interpretation

Nine NCCT markers of HE, including density (heterogeneous density, swirl sign, hypodensity, black hole sign, blend sign, fluid level) and shape (irregular shape, island sign, satellite sign) markers, were evaluated by two radiologists based on baseline CT images. Prior to the assessments, the radiologists familiarized themselves with the guidelines for interpretation provided by Morotti et al., which included diagnostic criteria and training cases for NCCT markers [1]. Two radiologists (X.Z and H.X) independently interpreted the NCCT markers without access to the clinical data or follow-up CT information of the patients. The diagnostic criteria and illustrative cases for NCCT markers were used as reference templates during the analysis. Consensus between the two investigators determined the presence or absence of each ICH marker. In cases of disagreement, a third radiologist (X.F) reviewed the images independently and the majority opinion was retained.

#### Lesion segmentation

Baseline and follow-up CT images were randomized and converted into Neuroimaging Informatics Technology Initiative (NIfTI) files before segmentation. Two experienced radiologists (X.Z and H.X) with 8 and 20 years of experience in neuroradiology respectively, performed lesion segmentation of ICH and IVH using ITK-SNAP software (version 3.8.0). ICH and IVH lesions were manually delineated slice-by-slice along the lesion margins on CT images. After segmentation, the individual slices were reconstructed into 3D representations of each region to calculate the volumes of ICH and IVH. A senior radiologist (L.S) confirmed all segmentations, and any disagreements were resolved through consensus.

#### Human reader prediction

Two physicians, J.S. and Z.Z., each with 5 and 20 years of experience in the field of neurology, respectively, independently assessed the risk of hematoma expansion based on three different definitions (RHE1, RHE2, CHE) using the clinical and imaging data provided in the study. They were instructed to estimate the probability of hematoma expansion on a scale ranging from 0 to 100, where 0 denoted no likelihood of expansion and 100 indicated a high probability of progressing toward hematoma expansion [2]. Notably, the predictions generated by the HENet model were not disclosed to the physicians during their evaluations.

#### Model establishment

HENet, a robust 2.5D DCNN model, was constructed based on the groundwork of 2D models and employed residual blocks in its design. The model used a pretrained ResNet101 as the feature extractor and was applied to three binary classification tasks, RHE1, RHE2 and CHE.

The HENet model employed a 2.5D input, primarily focused on the slice with the maximum lesion area along with its adjacent slices (two above and below (±2)). In contrast, the 2D model variant was constructed with the same architecture and hyperparameters as the 2.5D model, with the only difference being in the input configuration: it used only the slice with the maximum lesion area.

Each CT sequence was handled as an independent image, and the ROI was defined as the smallest rectangle encompassing the lesion areas across all slices. After cropping and resizing these ROIs, they were fed into parallel backbone networks to extract feature vectors of 2048-dimension. Max-pooling across slices produced a single feature vector of 2048-dimension, which was subsequently passed through a fully connected layer for binary classification.

To fully harness the benefits of the deep learning model, clinical (age, sex, onset to CT time, GCS score) and radiological features (ICH and IVH volume, ICH location, NCCT markers, SAH) were concatenated with deep learning features, resulting in a combined feature set. The overall architecture is illustrated in Figure 2.

#### Data preprocessing

The study utilized a range of essential techniques to address significant challenges inherent in medical image analysis. Pixel value truncation was conducted by constraining the pixel value intensities within the span of 45–100, thus diminishing the influence of extreme values and outliers. This normalization process standardized the datasets for improved reliability in subsequent analyses. Spatial normalization was initiated to handle voxel spacing incongruities across various volumes of interest (VOI). The fixed resolution resampling method was adopted for spatial normalization, ensuring a uniform voxel spacing of 1 mm × 1 mm × 1 mm and facilitating accurate comparisons and evaluations.

For data preparation, the maximum cross-section of the lesion region and the two upper and lower slices (±2) were extracted based on annotated ROI information, forming the minimum bounding rectangle for all subregions. During this cropping process, some samples might have been missing contextual information slices, such as when the maximum cross-section appeared at the bottom of the ROI. In these situations, the missing slices were filled using the maximum cross-section, leading to the creation of a 2.5D grayscale image dataset that contained three images. Following min-max transformation for normalization to achieve values in the range [−1, 1], each cropped subregion image was resized to 224 × 224 using nearest interpolation, ensuring compatibility with the input requirements of the models.

#### Model training process

To address overfitting during the training of the deep convolutional neural network (DCNN) models for all tasks, data augmentation techniques were implemented, which encompassed the following strategies: (1) randomly selecting the central slice of the 2.5D input from the top three slices with the largest lesion areas; (2) applying random translations within 10% and random scaling within the range [0.9, 1.1] with respect to the ROI; and (3) performing horizontal and vertical mirroring of the cropped ROI with a 50% probability.

The learning rate for the backbone part of the model is determined based on the minimum and maximum learning rates, denoted as $\eta_{\min}^{i}$ and $\eta_{\max}^{i}$, respectively. $\eta_{\min}^{i}$ was set to 0, and $\eta_{\max}^{i}$ was set to 0.01. The parameter $T_{i}$ represents the number of iteration epochs. Since the backbone part utilized pre-trained parameters, fine-tuning on it at $T_{cur}=\frac{1}{2}T_{i}$ were performed to ensure effective knowledge transfer. Therefore, the learning rate for the backbone part was computed as follows:

$$\eta_{t}^{task-spec}=\eta_{\min}^{i}+\frac{1}{2}\left(\eta_{\max}^{i}-\eta_{\min}^{i}\right)\left(1+\cos\left(\frac{T_{cur}}{T_{i}}\pi \right)\right)$$

The minimum learning rate, denoted as $\eta_{\min}^{i}$, was set to 0, while the maximum learning rate, denoted as $\eta_{\max}^{i}$, was set to 0.01. The parameter $T_{i}$ represented the number of iteration epochs. Since the backbone part of the model utilizes pre-trained parameters, fine-tuning on the backbone part at $T_{cur}=\frac{1}{2}T_{i}$ was performed to ensure effective transfer of knowledge. Consequently, the learning rate for the backbone part was determined as follows:

$$\eta_{t}^{backbone}=\left\{ \begin{array}{ll} 0 & \;\text{if}\;T_{cur}\leq \frac{1}{2}T_{i}\\ \eta_{\min}^{i}+\frac{1}{2}\left(\eta_{\max}^{i}-\eta_{\min}^{i}\right)\left(1+\cos\left(\frac{T_{cur}}{T_{i}}\pi \right)\right) & \;\text{if}\;T_{cur}>\frac{1}{2}T_{i} \end{array}\right.$$

The model underwent training for approximately 2967 iterations, equivalent to 90 epochs, with an early stopping round set at 32. The batch size was configured to 16, the SGD optimizer was employed. All training procedures were executed on an NVIDIA 4090 GPU, utilizing Onekey version 2.5.3 and PyTorch version 1.8.1. The procedure for data processing and training is illustrated in Figure S5.

#### 3D deep learning signature

The 3D model utilized the smallest enclosing cuboid that fully encompassed the ROI. To standardize input dimensions across patients, we resized each ROI to a fixed resolution of 48 × 48 × 48 voxels. For model development, we explored the resnet101 deep learning architectures. To ensure the model’s effectiveness across diverse patient cohorts, we adopted transfer learning by initializing the model with pre-trained weights from the ImageNet database. This approach enhanced the model’s adaptability to different datasets. A key component of our methodology was the careful tuning of the learning rate to achieve better generalization across datasets. We employed a cosine decay learning rate strategy, defined as follows:

$$\eta_{t}=\eta_{\min}^{i}+\frac{1}{2}\left(\eta_{\max}^{i}-\eta_{\min}^{i}\right)\left(1+\cos\left(\frac{T_{cur}}{T_{i}}\pi \right)\right)$$

where $\eta_{\min}^{i}=0$ is the minimum learning rate, $\eta_{\max}^{i}=0.01$ is the maximum learning rate, and $T_{i}=20$ represents the number of epochs in the iterative training process. Other critical hyperparameters included the use of Stochastic Gradient Descent (SGD) as the optimizer and softmax cross-entropy as the loss function.

#### Clinical signature

Machine learning models were developed using Clinical features. Hyperparameters were fine-tuned through 5-fold cross-validation and a Grid-Search optimization process. Logistic Regression was used for linear modeling, while ExtraTrees was implemented to capture complex feature interactions.

#### Visual explanations generation

Following the training and testing of the HENet model, visual explanations were conducted to gain insights into the specific image components that the model focuses on when making decisions. Grad-CAM was employed as the means to generate these visual explanations for each image patch.

Class localization maps were generated by visualizing the gradients flowing into the final convolutional layer of the network, which takes place immediately prior to the fully connected layers. This particular layer was chosen for its ability to preserve spatial information that is pertinent to the input image’s class, a characteristic that may otherwise be compromised in the fully connected layers.

Significantly, the Grad-CAM technique facilitates the generation of these maps without necessitating any alterations to the existing model architecture or additional model training.

### Quantification and statistical analysis

Categorical data were presented as absolute and relative frequencies. For continuous variables, normality was assessed using the Shapiro-Wilk test. When violated, continuous variables were reported as medians with interquartile ranges (IQRs). The chi-square test or Kruskal-Wallis test was employed to examine the differences in patient and hematoma characteristics among the three datasets.

Regarding model performance, in both the YY and LA datasets, the predictive performances of HENet Model, 2D Model, and physicians’ prediction were evaluated using receiver operating characteristic (ROC) curves. Additionally, calibration curve and decision curve analysis (DCA) were conducted to assess the calibration performance and clinical utility. The calibration ability of the HENet Model, 2D Model, and physicians’ prediction was assessed using the Hosmer-Lemeshow goodness-of-fit test.

In terms of model comparison, the performance of the HENet model was compared with that of the 2D model, physicians’ predictions, the clinical-only ExtraTrees model, and the 3D model using multiple discriminatory improvement measures: specifically, the increase in the area under the curve (AUC) assessed through the DeLong test, along with the integrated discrimination index (IDI) and continuous net reclassification index (NRI). The architectures of the clinical-only ExtraTrees model and the 3D model are detailed in the supplementary methods.

All statistical analyses were performed using R version 3.6.1 and Python 3.11.3.
