# Supplementary material for: Explainable CT-based deep learning model for predicting hematoma expansion including intraventricular hemorrhage growth
Source: iScience. 2025 Jun 13;28(7):112888. doi: 10.1016/j.isci.2025.112888 (PMC12246629; doi:10.1016/j.isci.2025.112888)
Supplement: Document S1. Figures S1–S5 and Tables S1–S11 [file mmc1.pdf]

iScience, Volume *28*

Supplemental information

Explainable CT-based deep learning model for predicting hematoma expansion including intraventricular hemorrhage growth

Xianjing Zhao, Zhengxiang Zhang, Juntao Shui, Hui Xu, Yulong Yang, Lequn Zhu, Lei Chen, Shixin Chang, Chunzhong Du, Zhenwei Yao, Xiangming Fang, and Lei Shi

Supplemental Materials

Figures S1–S5

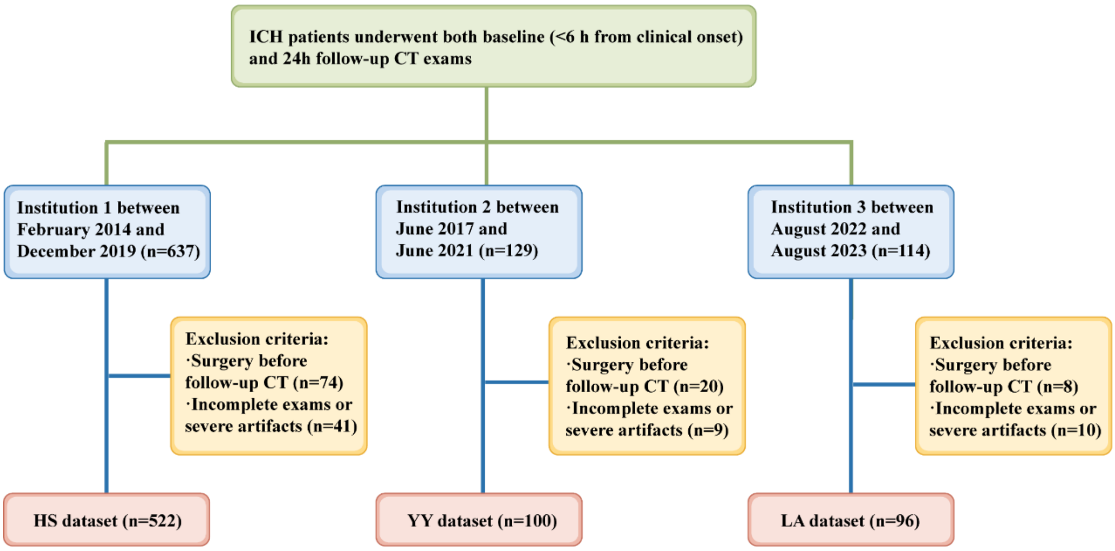

Figure S1 Patient Recruitment Process

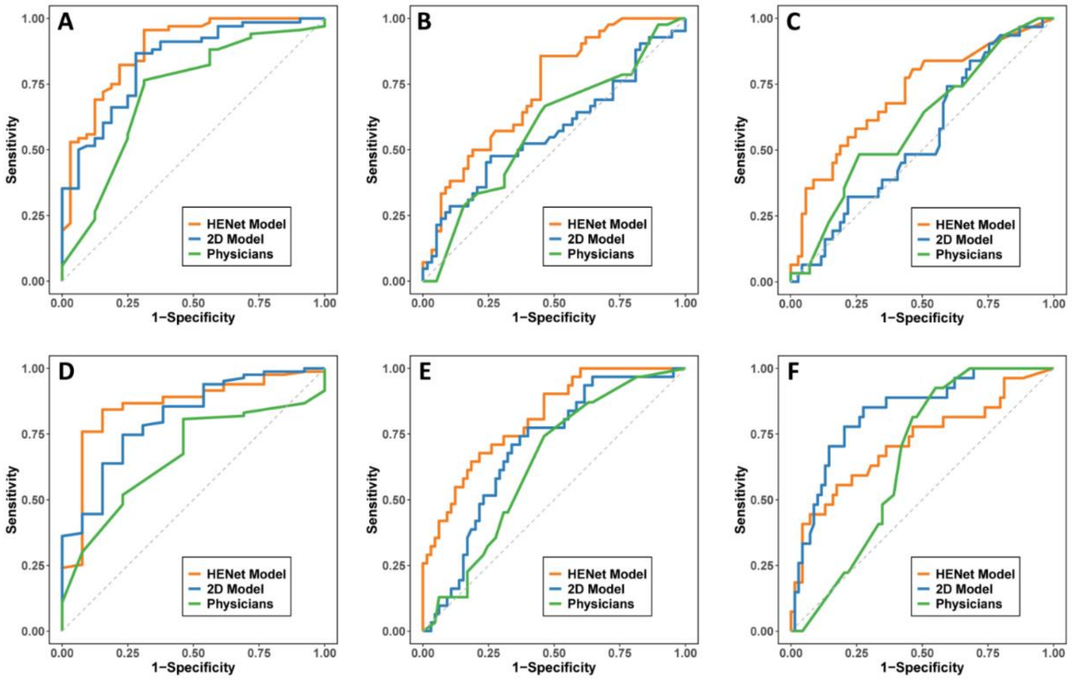

Figure S2: Receiver Operating Characteristic Curve Graphs for HENet Model, 2D

Model, and Physicians in Predicting Hematoma Expansion.

Graphs (A), (B), and (C) correspond to the Receiver Operating Characteristic (ROC) curves of HENet Model, 2D Model, and physicians predicting RHE1, RHE2, and CHE in the YY dataset, respectively. Graphs (D), (E), and (F) correspond to the ROC curves of HENet Model, 2D Model, and physicians predicting RHE1, RHE2, and CHE in the LA dataset, respectively.

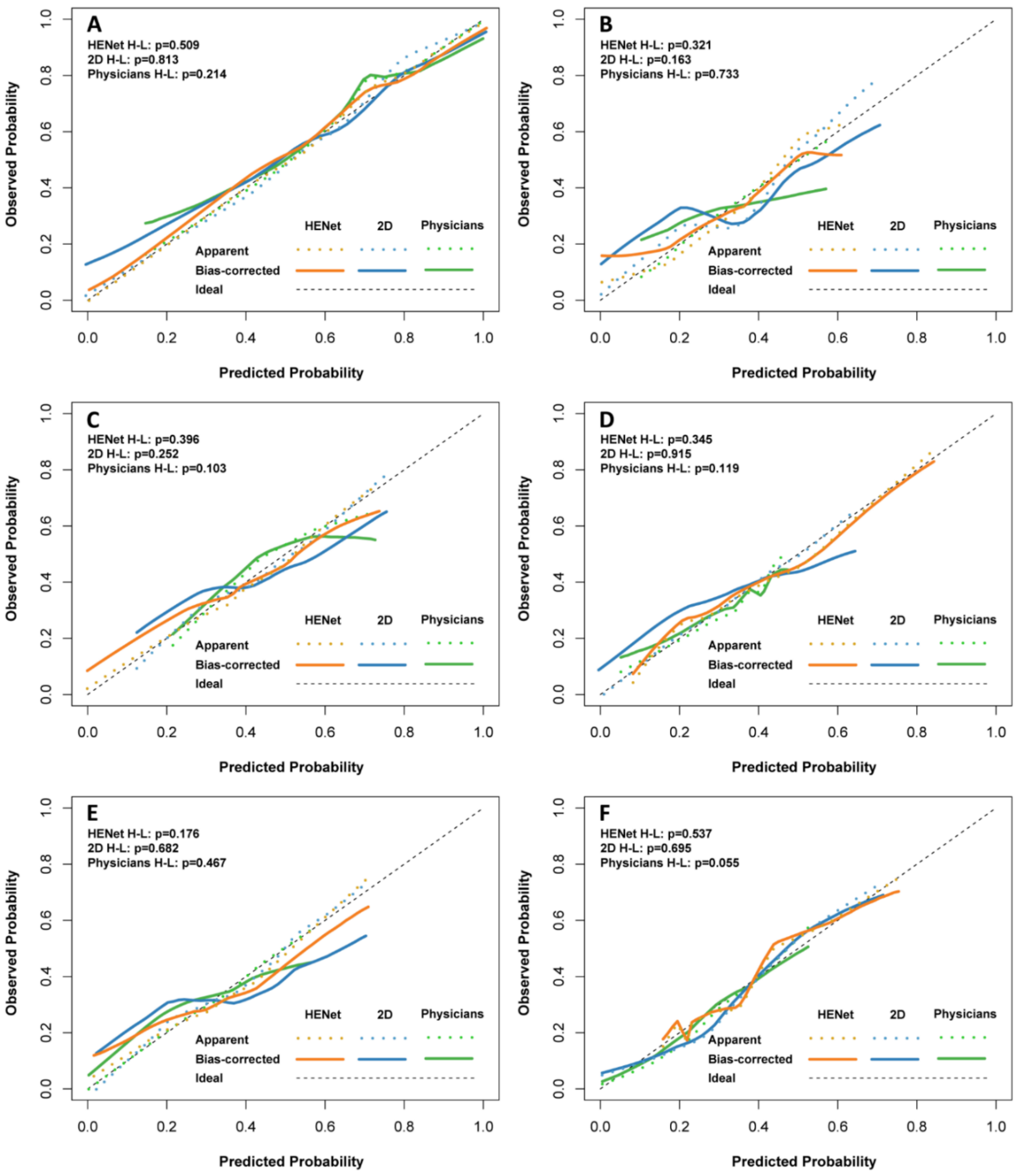

Figure S3: Calibration Curves for HENet Model, 2D Model, and Physicians in Predicting Hematoma Expansion.

Graphs (A), (C), and (E) represent the calibration curves of HENet Model, 2D Model, and physicians predicting RHE1, RHE2, and CHE in the YY dataset, respectively. Graphs (B), (D), and (F) represent the calibration curves of HENet Model, 2D Model,

and physicians predicting RHE1, RHE2, and CHE in the LA dataset, respectively.

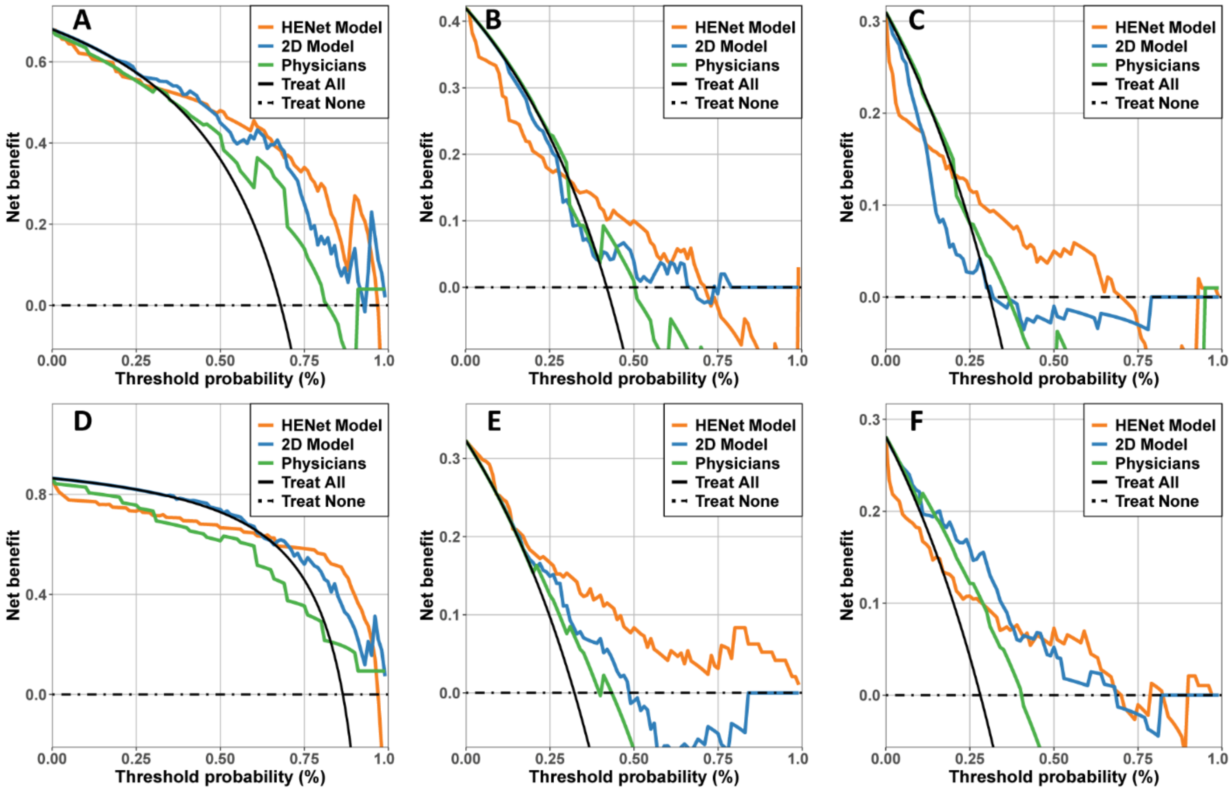

Figure S4: Decision curve analysis for HENet Model, 2D Model, and Physicians in Predicting Hematoma Expansion.

Graphs (A), (B), and (C) represent the decision curves of HENet Model, 2D Model, and physicians predicting RHE1, RHE2, and CHE in the YY dataset, respectively. Graphs (D), (E), and (F) represent the decision curves of HENet Model, 2D Model, and physicians predicting RHE1, RHE2, and CHE in the LA dataset, respectively.

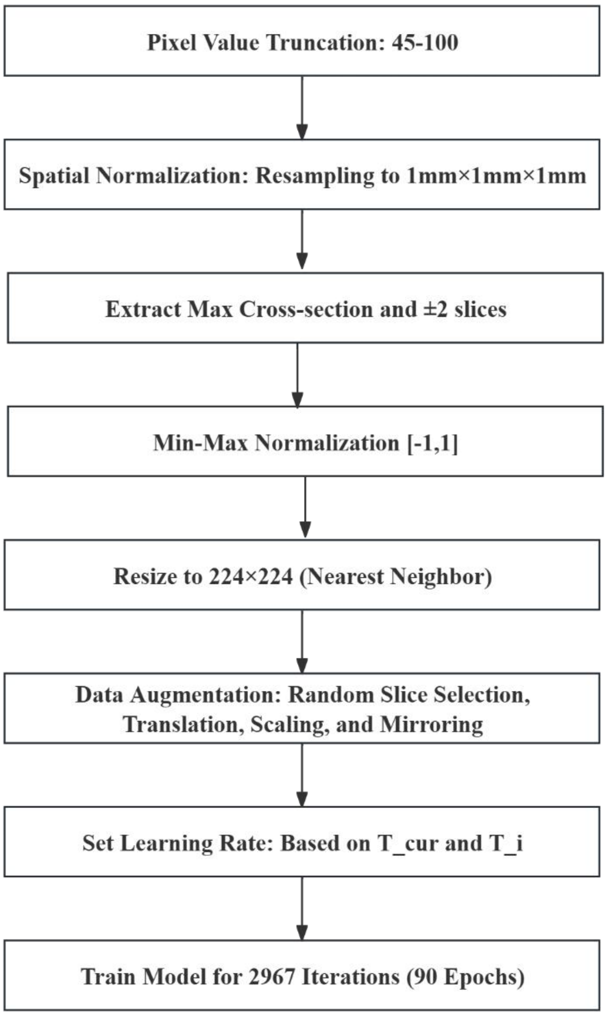

Figure S5: The Procedure for Data Processing and Training.

Tables S1–S11

Table S1: CT Scan Parameters for the HS, YY and LA Datasets

Dataset	HS Dataset		YY Dataset		LA Dataset	
CT scanner	GE Discovery CT750	Philips Brilliance iCT	SIEMENS SOMATOM Definition Flash	United Imaging uCT 510	United Imaging uCT 530	GE Optima CT680

Slice thickness (mm)	5	5	5	5	5	5
Tube current (mAs)	250	235	220	274	310	280
Tube voltage (KV)	120	120	120	120	120	120
Field of view (cm)	25	25	24	23	25	25
Matrix size	512 × 512	512 × 512	512 × 512	512 × 512	512 × 512	512 × 512

Table S2: Intraclass Correlation Coefficient of Hematoma Expansion Predicted by Two Physicians.

Outcome	Dataset	Intraclass Correlation Coefficient
RHE1	YY	0.773
	LA	0.789
RHE2	YY	0.761
	LA	0.742
CHE	YY	0.773
	LA	0.827

Table S3: Performance of Physicians' Prediction in Hematoma Expansion

Outcome	Dataset	AUC	95% CI	Accuracy	Sensitivity	Specificity	PPV	NPV
RHE1	YY	0.717	0.590 - 0.820	0.710	0.838	0.438	0.760	0.560
	LA	0.666	0.509 -	0.771	0.807	0.538	0.918	0.304

			0.814					
RHE2	YY	0.583	0.478 - 0.703	0.570	0.405	0.690	0.486	0.616
	LA	0.636	0.526 - 0.741	0.604	0.452	0.677	0.400	0.721
CHE	YY	0.600	0.485 - 0.712	0.660	0.484	0.739	0.455	0.761
	LA	0.654	0.541 - 0.756	0.583	0.519	0.609	0.341	0.764

Abbreviations: AUC, area under the curve; CHE, conventional hematoma expansion; NPV, negative predictive value; PPV, positive predictive value; RHE1, revised hematoma expansion definition one; RHE2, revised hematoma expansion definition two.

Table S4: Performance of 2D Model in Predicting Hematoma Expansion

Outcome	Dataset	AUC	95% CI	Accuracy	Sensitivity	Specificity	PPV	NPV
RHE1	YY	0.836	0.752 - 0.919	0.820	0.868	0.719	0.868	0.719
	LA	0.811	0.688 - 0.935	0.750	0.747	0.769	0.954	0.323
RHE2	YY	0.573	0.455 - 0.691	0.630	0.476	0.741	0.571	0.662
	LA	0.697	0.589 - 0.805	0.656	0.774	0.609	0.480	0.848
CHE	YY	0.546	0.427 - 0.665	0.480	0.839	0.324	0.356	0.815
	LA	0.832	0.743 -	0.760	0.852	0.735	0.548	0.926

		0.921						

Abbreviations: AUC, area under the curve; CHE, conventional hematoma expansion; NPV, negative predictive value; PPV, positive predictive value; RHE1, revised hematoma expansion definition one; RHE2, revised hematoma expansion definition two.

Table S5: Comparative Analysis of HENet Model and Physicians' Prediction in Predicting Hematoma Outcome

Outcome	Dataset	Delong Test		Continuous NRI		IDI Analysis	
		△AUC（95%CI）	*P* Value	NRI（95%CI）	*P* Value	IDI（95%CI）	*P* Value
RHE1	YY	0.16(0.06 - 0.27)	0.003	0.92(0.57 - 1.27)	<0.001	0.36(0.23 - 0.49)	<0.001
	LA	0.19(0.01 - 0.37)	0.037	1.05(0.69 - 1.41)	<0.001	0.43(0.25 - 0.62)	<0.001
RHE2	YY	0.14(0 - 0.29)	0.046	0.46(0.09 - 0.83)	0.015	0.15(0.01 - 0.29)	0.035
	LA	0.18(0.03 - 0.32)	0.015	0.76(0.37 - 1.15)	<0.001	0.23(0.06 - 0.39)	0.006
CHE	YY	0.11(-0.03 - 0.26)	0.132	0.33(-0.05 - 0.71)	0.088	0.13(0.00 - 0.26)	0.047
	LA	0.06(-0.11 - 0.23)	0.472	0.03(-0.37 - 0.43)	0.894	0.08(-0.12 - 0.27)	0.431

Abbreviations: CI, confidence intervals; IDI, integrated discrimination improvement; NRI, net reclassification improvement.

Table S6: Comparative Analysis of HENet Model and 2D Model in Predicting Hematoma Outcome

Outcome	Dataset	Delong Test		Continuous NRI		IDI Analysis	
		△AUC（95%CI）	*P* Value	NRI（95%CI）	*P* Value	IDI（95%CI）	*P* Value
RHE1	YY	0.05(-0.03 - 0.13)	0.264	0.89(0.55 - 1.24)	<0.001	0.25(0.14 - 0.37)	<0.001
	LA	0.04(-0.04 - 0.13)	0.347	0.92(0.56 - 1.28)	<0.001	0.32(0.20 - 0.44)	<0.001
RHE2	YY	0.15(0.03 - 0.28)	0.014	0.62(0.26 - 0.98)	<0.001	0.16(0.05 - 0.28)	0.006
	LA	0.11(-0.01 - 0.24)	0.081	0.80(0.40 - 1.19)	<0.001	0.22(0.10 - 0.34)	<0.001
CHE	YY	0.26(0.07 - 0.45)	0.006	0.75(0.35 - 1.14)	<0.001	0.21(0.09 - 0.34)	<0.001
	LA	-0.12(-0.27 - 0.04)	0.147	0.18(-0.26 - 0.62)	0.420	0.04(-0.12 - 0.21)	0.601

Abbreviations: CI, confidence intervals; IDI, integrated discrimination improvement; NRI, net reclassification improvement.

Table S7 Performance (the F1 Score, Precision, and Recall) of HENet Model in Predicting Hematoma Expansion

Outcome	Dataset	Precision	Recall	F1 Score
RHE1	YY	0.867	0.956	0.909
	LA	0.972	0.843	0.903
RHE2	YY	0.580	0.857	0.692
	LA	0.600	0.677	0.636
CHE	YY	0.439	0.807	0.568
	LA	0.556	0.556	0.556

Abbreviations: CHE, conventional hematoma expansion; RHE1, revised hematoma expansion definition one; RHE2, revised hematoma expansion definition two.

Table S8: Performance of 3D Model in Predicting Hematoma Expansion

**Outcome**	**Dataset**	**AUC**	**95% CI**	**Accuracy**	**Sensitivity**	**Specificity**	**PPV**	**NPV**	**Precision**	**Recall**	**F1 Score**
RHE1	YY	0.826	0.742-0.909	0.800	0.765	0.875	0.929	0.636	0.929	0.765	0.839
	LA	0.723	0.571-0.876	0.823	0.867	0.538	0.923	0.389	0.923	0.867	0.894
RHE2	YY	0.430	0.330	0.450	0.976	0.069	0.432	0.800	0.432	0.976	0.599

			- 0.531								
	LA	0.552	0.436 - 0.668	0.490	0.903	0.292	0.378	0.864	0.378	0.903	0.533
CHE	YY	0.534	0.410 - 0.659	0.530	0.645	0.478	0.357	0.750	0.357	0.645	0.460
	LA	0.597	0.464 - 0.730	0.677	0.556	0.725	0.441	0.806	0.441	0.556	0.492

Table S9: Comparative Analysis of HENet Model and 3D Model in Predicting Hematoma Outcome

Outcome	Dataset	Delong Test		Continuous NRI		IDI Analysis	
		△AUC（95%CI）	*P* Value	NRI（95%CI）	*P* Value	IDI（95%CI）	*P* Value
RHE1	YY	0.06 (-0.04 - 0.15)	0.245	0.83（0.49 - 1.18)	<0.001	0.34（0.20 - 0.47)	<0.001
	LA	0.13 (-0.06 - 0.32)	0.18	0.93（0.57 - 1.29)	<0.001	0.42（0.22 - 0.61)	<0.001
RHE2	YY	0.30（0.15	<0.00	0.47（0.09	0.015	0.22（0.10	<0.00

		- 0.44)	1	- 0.86)		- 0.33)	1
	LA	0.26 (0.12 - 0.40)	<0.001	0.80 (0.41 - 1.20)	<0.001	0.32 (0.20 - 0.44)	<0.001
CHE	YY	0.18 (0.02 - 0.33)	0.023	0.78 (0.38 - 1.17)	<0.001	0.21 (0.08 - 0.35)	0.002
	LA	0.12 (-0.07 - 0.31)	0.222	0.59 (0.17 - 1.02)	0.007	0.23 (0.09 - 0.37)	0.001

Abbreviations: CI, confidence intervals; IDI, integrated discrimination improvement; NRI, net reclassification improvement.

Table S10: Performance of ExtraTrees Model in Predicting Hematoma Expansion

**Outcome**	**Dataset**	**AUC**	**95% CI**	**Accuracy**	**Sensitivity**	**Specificity**	**PPV**	**NPV**	**Precision**	**Recall**	**F1 Score**
RHE1	YY	0.581	0.471 - 0.692	0.71	0.941	0.219	0.719	0.636	0.719	0.941	0.815
	LA	0.565	0.439 - 0.692	0.365	0.277	0.923	0.958	0.167	0.958	0.277	0.43
RHE2	YY	0.482	0.378 - 0.586	0.58	0.071	0.948	0.5	0.585	0.5	0.071	0.125

	LA	0.716	0.605-0.826	0.76	0.516	0.877	0.667	0.792	0.667	0.516	0.582
CHE	YY	0.438	0.334-0.542	0.31	1	0	0.31	0	0.31	1	0.473
	LA	0.648	0.532-0.765	0.771	0.407	0.913	0.647	0.797	0.647	0.407	0.5

Table S11: Comparative Analysis of HENet Model and ExtraTrees Model in Predicting Hematoma Outcome

Outcome	Dataset	Delong Test		Continuous NRI		IDI Analysis	
		△AUC（95%CI）	*P* Value	NRI（95%CI）	*P* Value	IDI（95%CI）	*P* Value
RHE1	YY	0.30 (0.17 - 0.43)	<0.001	1.07 (0.74 - 1.40)	<0.001	0.53 (0.36 - 0.71)	<0.001
	LA	0.29 (0.13 - 0.45)	0.001	1.24 (0.89 - 1.59)	<0.001	0.54 (0.36 - 0.71)	<0.001
RHE2	YY	0.25 (0.10 - 0.39)	0.001	0.59 (0.21 - 0.97)	0.002	0.21 (0.09 - 0.33)	<0.001
	LA	0.10 (-0.03 - 0.22)	0.127	0.83 (0.44 - 1.22)	<0.001	0.28 (0.16 - 0.40)	<0.001
CHE	YY	0.27 (0.11 - 0.44)	0.001	0.64 (0.24 - 1.04)	0.002	0.24 (0.10 - 0.37)	<0.001
	LA	0.07 (-0.10 - 0.23)	0.421	0.62 (0.20 - 1.05)	0.004	0.20 (0.06 - 0.33)	0.004

Abbreviations: CI, confidence intervals; IDI, integrated discrimination improvement; NRI, net reclassification improvement.
